# The determinants of receiving social care in later life in England

**DOI:** 10.1017/S0144686X1300072X

**Published:** 2013-09-23

**Authors:** A. VLACHANTONI, R. J. SHAW, M. EVANDROU, J. FALKINGHAM

**Affiliations:** * EPSRC Care Life Cycle, University of Southampton, UK.; † ESRC Centre for Population Change, University of Southampton, UK.; ‡ Centre for Research on Ageing, University of Southampton, UK.

**Keywords:** older people, social care, informal care, support, English Longitudinal Study of Ageing (ELSA)

## Abstract

Demographic change and policy changes in social care provision can affect the type of social care support received by older people, whether through informal, formal state or formal paid-for sources. This paper analyses the English Longitudinal Study of Ageing data (wave 4) in order to examine the relationship between demographic and socio-economic characteristics, and the receipt of support from different sources by older people who report difficulty with daily activities. The research outlines three key results with implications for the future organisation of social care for older people. Firstly, the number of instrumental activities of daily living (IADLs) an older person reports having difficulty with, followed by the number of activities of daily living (ADLs) are the strongest determinants of receiving support from any source. Secondly, there are significant gender differences in the factors associated with receiving support from different sources; for example, physical health is a strong determinant of informal support receipt by men, while mental health status is a strong determinant of informal support receipt by women. Finally, the research shows that different kinds of impediments in everyday life are associated with receiving support from different sources. This ‘link’ between particular types of difficulties and support receipt from particular sources raises questions about the way social care provision can or should be organised in the future.

## Introduction

The need for support in later life for individuals living in the community can be met through a combination of contributions from the informal, formal state and formal paid-for sector, however, informal networks of family and friends constitute the cornerstone of care provision for older people, and a more common source of support for those who have children compared to formal state or formal paid-for sources of care (Grundy and Read 2012).

In the English context, which is the focus of this paper, research has shown that 32 per cent of men and 41 per cent of women aged 50 and over relied exclusively on informal sources for support in later life, while 5 per cent of men and 8 per cent of women relied on formal sources (state or paid-for) for such support (Breeze and Stafford 2010). At the same time as receiving support in later life, older people significantly contribute to the provision of informal care to their spouse or other family members, or neighbours. For example, the 2001 UK Census indicated that approximately 1.24 million men and 1.56 million women over the age of 50 provide unpaid care to sick/disabled persons, with approximately half of these concentrated among those aged between 50 and 59 (Office for National Statistics 2003). Existing research shows that informal support tends to be provided by women across the lifecourse, except in older age (*i.e.* 70 years and over), when men are more likely to care for their spouses largely as a result of gender differences in men's and women's marital status in later life (Dahlberg, Demack and Bambra 2007; Del Bono, Sala and Hancock 2009). The majority of care provided by people aged 50 and over takes up to 19 hours per week (56 per cent), but Hyde and Janovic (2004) found that about one-quarter of older carers devoted at least 50 hours a week to caring.

Demographic changes across the developed world can directly affect the demand for health and social care. In the United Kingdom (UK) the growth in the number of the ‘oldest old’ is often cited as an indicator of the increase in high-support needs among the older population, and the concomitant increase in demand on support services (Falkingham *et al.* 2010). The number of people aged 65 and over in the UK is projected to increase from almost 10 million in 2008 to about 13 million by 2021, and almost 16 million in 2031 (Office for National Statistics 2009). However, the fastest growth within the older population is expected among those aged 85 and over, who in the UK constituted 1.4 million people in 2009, and are expected to reach 3.5 million, or 5 per cent of the total population, by 2034 (Office for National Statistics 2010), raising policy concerns about the future provision of health and social care.

At the same time, demographic change may also affect the supply of informal care towards older people. On the one hand, a rise in the absolute number of older people, many of whom are healthy and can continue to provide informal care to other persons, points to a steady supply of informal care in later life which can have positive consequences for both the carer and the cared-for person. For example, Thomas (2010) used data from the United States of America (USA) and showed that the provision of support by persons aged 50 and over was beneficial to their wellbeing, taking into account the type of support, the amount of support and the relationship to the person receiving the support. On the other hand, Pickard *et al.* (2000) summarise a number of demographic changes which may work in the opposite direction, including a rise in divorce rates, a low fertility rate which results in smaller family sizes, the rising incidence of childlessness, rising labour market participation among women, the rising incidence of older people living alone in later life – particularly women, the changing nature of kinship obligations and finally the changing preferences in terms of social care receipt of successive cohorts of older people. The way in which demographic change will interact with the supply of informal care in the future has direct implications for the level and nature of informal care which can be expected to be provided and received.

Demographic change has been accompanied in many developed countries, including the UK, with changes in the policy context of social care provision, which can adversely affect the capacity of local councils to meet the needs of older people. Yeandle, Kröger and Cass (2012), for example, compared policy reforms in Australia, England and Finland, and argued that the experience of greater privatisation, and the construction of more individualised and personalised services, is connected with a greater reliance on informal care provision in these countries. In the British context, since the early 1990s one of the key trends in social care provision has been the targeting of resources to those whose needs are deemed ‘substantial’ or ‘critical’ (NHS Information Centre 2010), while older people with moderate needs are often supported by the informal or paid-for sources, or a combination of the two. Part of the explanation for such intensification of social services lies with the increasing cost of providing long-term care: for instance, between 2004–05 and 2008–09, the cost of providing nursing care (in pounds per person per week) increased by 15.5 per cent, while the equivalent cost of providing home care increased by 26 per cent.^1^ The statutory sector's capacity to respond to increasing demand is hampered by the recent economic crisis, for instance Humphries and colleagues noted that by 2007, approximately 72 per cent of all councils had restricted their eligibility threshold to individuals with ‘substantial’ or ‘critical’ need (Humphries, Forder and Fernandez 2010). Exploring the connections between demographic and socio-economic characteristics of older people, their report of difficulty with day-to-day activities and their receipt of support from different sources is an important step towards our understanding of the role each source plays in safeguarding the wellbeing of older people.

Focusing on the English context, this paper investigates the factors associated with the receipt of social care support in later life from informal, formal state or formal paid-for sources for individuals living in the community. As such, the paper aims to contribute to the academic literature which explores the effect of demographic and socio-economic predictors on an older person's receipt of support from different sources. The rest of the paper contextualises the research in the relevant academic literature and our conceptual framework, discusses the data and methodology used, presents the results, and discusses the results in the light of existing research and social policy design in the area of social care.

## The receipt of social care support in later life

The academic literature which this paper contributes to departs from the premise that an individual's physical and mental health status is associated with the amount and type of social care support required in later life (Breeze and Stafford 2010). However, the level and nature of support required may vary depending on a host of characteristics, including an individual's demographic and socio-economic characteristics, and the extent to which individuals can use technologies in their home environment to compensate for disabilities (Costa-Font 2008; Graciani *et al.* 2004).

An individual's marital status, living arrangements and whether they have children are key indicators of the extent to which they can expect to receive informal support from family in later life. In this respect, the literature points to changes in marital status and living arrangements over the lifecourse which can adversely affect an individual's physical and mental health status, as well as the availability of informal support in later life (Blomgren *et al.* 2010; Glaser *et al.* 2008). The majority of such literature is context-specific, reflecting the norms and values of a society towards the provision of social care by the state or by younger generations. Finch (1989) and Finch and Mason's (1993) seminal works on the negotiations within English families about caring responsibilities indicated that although relationships between elderly parents and adult children are founded upon a sense of obligation, nevertheless such feelings have limits when it comes to the provision of care, and one's sense of obligation may also be affected by their preferences. In a different country context, and reflecting on the development of the state system of care provision, Daatland (1990) conducted interviews with people aged 70 and over in Norway, and found that older people preferred the use of public services for practical or personal support in the long term, but turned to their children's informal support in order to satisfy short-term needs.

Literature focusing on particular country contexts also explores the social or economic resources which are necessary for accessing support from a particular source, and the link between particular types of need and the receipt of support from particular sources. Such literature, for example on the British (Glaser *et al.* 2008) and Swedish context (Larsson and Silverstein 2004), has found that indicators of an individual's higher socio-economic status are negatively associated with the receipt of informal support from family members, or formal state support from social services, and positively associated with the receipt of paid-for support from the private market. Comparative literature in this area is scarce, and has either found little evidence for the link between a higher socio-economic status and the receipt of informal or formal support (Motel-Klingebiel, Tesch-Roemer and von Kondratowitz 2005), or has provided evidence of a differential ‘behaviour’ of socio-economic factors in different countries (Broese van Groenou *et al.* 2006).

Finally, a part of the literature in this area is concerned with the extent to which support from one source may be ‘substituted’ by support from another source. On one hand, there is evidence for the decline of informal support in the face of increasing formal support (Stabile, Laporte and Coyte 2006; Weissert, Matthews Cready and Pawelak 1988), while another part of the literature argues that the introduction of more formal care supplements, rather than substitutes, informal care (Kuenemund and Rein 1999), and finally Mentzakis, McNamee and Ryan (2009) note that such effects depend on the specific task for which care is received.

**Figure 1.**
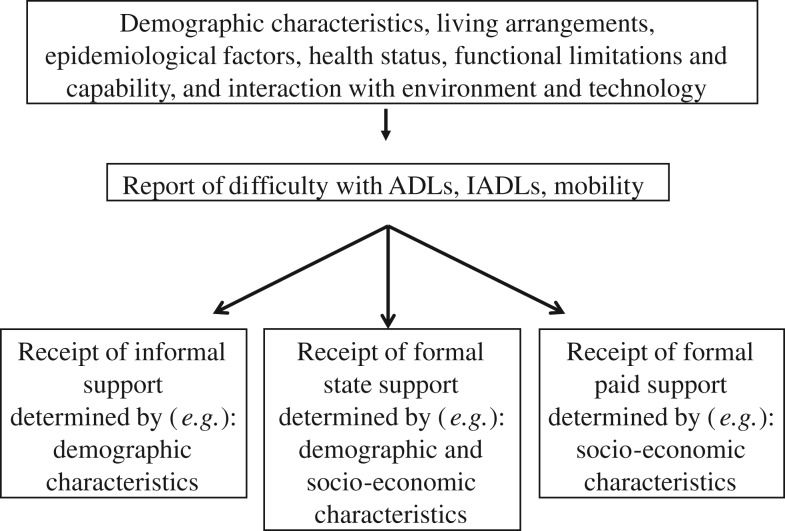
Conceptualising the receipt of social care support in later life. *Notes*: ADLs: activities of daily living. IADLs: instrumental activities of daily living.

Figure 1 outlines the conceptual framework of the research and proposes that an individual's need for care, which acts as a prerequisite for receiving social care support, may result from a range of factors, including an individual's physical and mental health status, their living arrangements, as well as the extent to which they can use technologies in order to adjust their living environment. An individual's need for care may be determined in various ways, *e.g.* through self-reported measures or through information collected from professionals or carers who support individuals in their day-to-day activities. In this paper, we use three different self-reported measures of difficulty with day-to-day activities and mobility as predictors of the source of support received by older people, and these measures are explained in the next section in greater detail. Individuals may receive social care support from the informal, formal state or formal paid-for sector, or any combination of the three. Drawing upon the existing literature in this area, we hypothesise that particular characteristics of an individual may be associated with the receipt of social care support from particular sources. For example, demographic characteristics, such as an individual's gender, marital status or whether they have children, may be stronger determinants of receiving support from informal sources, and, conversely, socio-economic characteristics may be more closely linked with an individual's receipt of formal paid support, as such factors reflect their ability to purchase such support. Finally, the receipt of state support may be associated with a combination of demographic and socio-economic characteristics, which are taken into account by state providers when assessing individuals for their eligibility to state support.

## Data and methodology

The research uses data from wave 4 of the English Longitudinal Study of Ageing (ELSA), which is a longitudinal survey of people aged 50 and over, who live in private households in England. The ELSA sample has been drawn from respondents to the Health Survey for England (HSE), which is an annual cross-sectional household survey which collects a wide range of health data and biometric measures. Each HSE sample is drawn using a two-stage sampling strategy, which involves a selection based on postcodes selected from the Postcode Address File and a random selection of households from a fixed number of addresses covering each postcode sector. As a result, the HSE is nationally representative of private households. There is a potential loss of representativeness before the ELSA data are drawn from HSE data due to non-response to HSE, refusal to be re-contacted, attrition between HSE and ELSA, and the exclusion of individuals living in institutions such as residential and nursing homes. However, such factors have been partly mitigated by weights in the ELSA.

The sample employed in this analysis focuses on core respondents of the ELSA dataset who are aged 65 and over and who had no missing information in their report of difficulty with at least one activity of daily living (ADL; *e.g.* dressing), instrumental activity of daily living (IADL; *e.g. doing* housework) or mobility (*e.g.* walking 100 yards), totalling 3,395 individuals. The tool of ADLs was developed in the 1960s, and is used in order to evaluate an individual's ability to perform functional activities independently (Katz *et al.* 1963). ADLs refer to basic functional abilities, such as bathing or dressing, whereas IADLs, such as managing one's finances, are located at a higher level of functioning, might require mental and/or physical capacity, and can diminish earlier than ADLs (Lawton and Brody 1969). Individuals who responded to the survey by proxy were not included in the analysis. Sub-samples have then been used to study particular aspects of social care receipt. The report of difficulty with at least one ADL, IADL or mobility is a key threshold in the ELSA dataset, as it determines whether the respondent will be asked further questions regarding the receipt of support from different sources, such as informal, state or paid-for services.

The research distinguishes between different sources of support in later life as follows, taking into account previous research (Breeze and Stafford 2010). Informal support refers to the support received from one's spouse (husband, wife or civil partner), partner, son, daughter, sister, brother, other relative, friend or neighbour. State support refers to the support received from a home-care worker or a district nurse, and finally paid-for support refers to the support received from privately paid sources. The category of ‘other support’ includes members of staff at a care/nursing home, which the dataset did not specify whether it was paid for privately or by the state. The ELSA questionnaire collects detailed information on the respondents' health status if they are aged 65 and over, and also enquires about the source of support received by the respondent only if they have reported at least one difficulty with an ADL, IADL or mobility task. As a result, the bivariate analysis cross-tabulated, firstly, the report of a difficulty with an ADL, IADL or mobility task with the respondent's gender and age group, and secondly, the report of a difficulty with an ADL, IADL or mobility task with the receipt of support from an informal, formal state or formal paid-for source.

The multivariate analysis employs logistic regressions, and models were run for three separate binary outcome variables corresponding to the receipt of support from informal, state and paid-for sources. The final models for each outcome were selected using a sequential model-building process, with model fitness being based on log-likelihood ratio tests. The emphasis on gender differences in the receipt of informal support within the existing literature (Young, Grundy and Jitlal 2006) was reflected in the significance of the gender variable within the model for both men and women (not shown here). As a result, the logistic regression model for individuals receiving informal support was run separately for men and for women in order to explore gender differences in the strength of the predictors. Initially, variables were allocated to one of seven potential categories of independent variables, which operationalise concepts emanating from the literature and are indicated in the conceptual framework of the research (Figure 1). Variables which were not independently associated with the outcomes within the same group were excluded at this stage, before the sequential modelling process was started. As the log-likelihood ratio for models needed to be comparable, a complete case analysis was conducted using all the variables to be used in the models. Starting with a base model containing demographic variables, variables from subsequent categories were then added to the model, and those variables which significantly improved the model fit were retained before the next category of variables was added, and the process was repeated until the final model was produced.

The seven categories include: *demographic variables* (gender, age group, legal marital status or co-habitation, having any children in the household, number of children outside the household and number of household members); *socio-economic variables*^2^ (benefit unit equivalised income and wealth,^3^ access to a car, housing tenure); *physical health variables* (self-reported general health, self-reported eyesight, self-reported hearing, self-reported pain, and doctor's diagnosis of arthritis, chronic lung disease, Parkinson's disease or high blood pressure); *mental health variables* (doctor's diagnosis of depression or dementia, number of errors in orientation in time); *disability/functional limitations variables* (self-reported number of mobility limitations, ADLs and IADLs one has difficulty with, difficulty with walking a quarter of a mile, report of limiting long-standing illness (LLSI); *environment/technology variables* (self-report of home with an adaptation (*e.g.* hand rails^4^), retirement housing, current use of cane/walking stick, zimmer frame/walker, manual or electric wheelchair, buggy/scooter, personal alarm or elbow crutches); and *receipt of support/use of services variables* (self-report of receipt of informal, state, paid-for or ‘other’ support, whether respondent has ever attended a lunch club or day care centre, has ever used meals-on-wheels, and whether respondent is currently using the services of an occupational therapist/physiotherapist, chiropodist or is engaging in exercise therapy).

## Results

**Figure 2.**
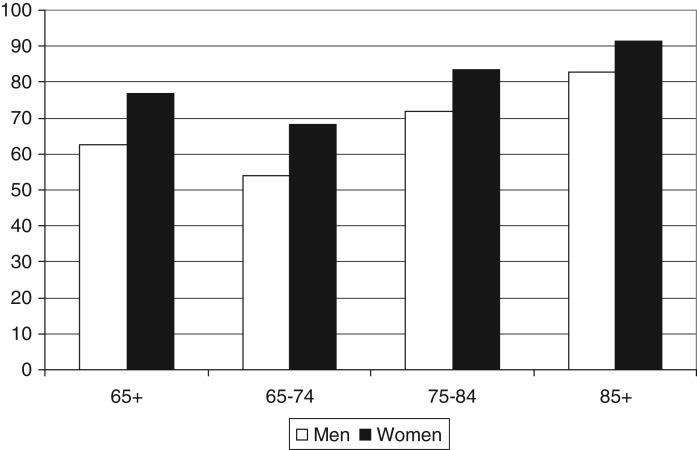
Percentage of older people who report a difficulty with activities of daily living, instrumental activities of daily living or mobility, by age group and gender, England, 2008. *Source*: English Longitudinal Study of Ageing wave 4, authors' calculations. *Significance*: 65+: *χ*^2^=118.9565; df=1; *p*<0.0001; 65–74: *χ*^2^=63.0404; df=1; *p*<0.0001; 75–84: *χ*^2^=28.5425; df=1; *p*<0.0001; 85+: *χ*^2^=7.4541; df=1; *p*=0.0134.

The paper first presents results of the bivariate analysis, followed by results of the multivariate analysis. Figure 2 shows that the report of difficulty with an ADL, IADL or mobility task increases in line with age, and there are significant gender differences, for example 62 per cent of men aged 65–74 report a difficulty, compared to 77 per cent of women in the same age group.

**Figure 3.**
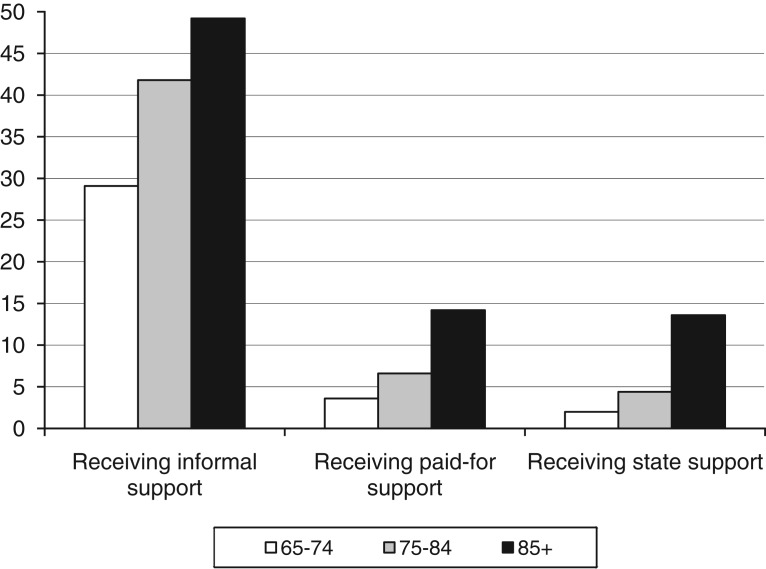
Among older people who report a difficulty, percentage who receive support, by age group and source of support, England, 2008. *Note*: The percentages in each source of support add to more than 100 per cent, as some individuals received support from more than one source. *Source*: English Longitudinal Study of Ageing wave 4, authors' calculations, weighted percentages. *Significance*: Informal care: *χ*^2^=88.7332; df=2; *p*<0.0001; paid-for care: *χ*^2^=74.5647; df=2; *p*<0.0001; state care: *χ*^2^=118.1830; df=2; *p*<0.0001.

Figure 3 shows the source of support amongst older people who reported a difficulty. The first point to note is the primacy of informal support receipt compared to support from paid-for or state sources. For example, 29 per cent of people aged 65–74 who report a difficulty receive informal support, compared to 4 per cent of people in this age group who receive paid-for care and 2 per cent who receive state care. The proportion of older people who receive support from different sources increases by age: for example almost half of all people aged 85 and over who report a difficulty receive support from informal sources, compared to 42 per cent of people aged 75–84, and 29 per cent of the youngest age group.

**Figure 4.**
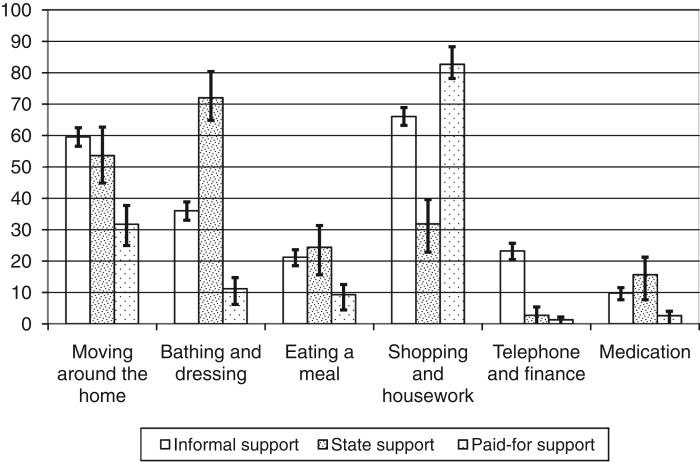
Among older people who report a difficulty and receive support, percentage receiving support by activity and source of support, England, 2008. *Notes*: The percentages in each activity add to more than 100 per cent, as certain individuals received support for more than one activity. The extending bars reflect the standard errors of each estimate. *Source*: English Longitudinal Study of Ageing wave 4, authors' calculations, weighted percentages.

Among older people who report a difficulty and receive support, Figure 4 shows the percentage receiving support by the activity and source of support. The figure uses three examples of ADLs (moving; bathing; eating) and three examples of IADLs (shopping, housework, garden work; making telephone calls; receiving medication) in order to explore the activities for which older people receive support from different sources. These examples of activities were chosen for illustration, as they produced sufficiently strong sample counts for analytical purposes. Figure 4 shows a clear divide in the source of support received for particular kinds of activities, with state support more likely to be received for ADLs, and informal support, and to a lesser extent paid-for support, more likely to be received for IADLs. For example, amongst those who report a difficulty with bathing and dressing, 71 per cent receive state support, while amongst those who report difficulty with moving around the house, 59 per cent receive informal support.

The next part of this section turns to explore the determinants of receiving support from informal, state or paid-for sources, first discussing the development of the model and then the results of the analysis. Tables 1–4 present the final models showing the determinants of receiving informal support for all those who have reported at least one difficulty with an ADL, IADL or mobility task (separately for men and women), formal state support and formal paid-for support (significant results are shown in bold).

### The determinants of receiving informal support for men

The receipt of informal support for men is associated with higher age, having a partner/spouse, reporting a lung disease, reporting difficulties with mobility, ADLs, IADLs, reporting a LLSI, using a walking stick and using an occupational therapist. Reporting difficulty with a high number of IADLs is the strongest predictor of receiving informal support for men; the odds of receiving informal support amongst men experiencing difficulty with between five and nine IADLs are 31 times the odds of men who report experiencing no difficulty in performing IADLs (Table 1). On the other hand, among men reporting difficulties, those whose home has undergone some adaptation, and those who are in receipt of paid-for care, are less likely to be receiving informal support.

**Table 1.** The determinants of receiving informal support for men (final model)

	Unweighted N (base sample)	Weighted %	Odds ratio	Lower 95% CI	Upper 95% CI	*p*
Age group:						
65–69 (Ref.)	301	24.41	1			
70–74	363	25.82	0.98	0.58	1.67	0.945
75–79	244	22.23	**2.16**	1.24	3.74	0.006
80–84	181	16.38	**2.65**	1.50	4.66	0.001
85–89	82	7.74	1.06	0.49	2.33	0.873
90+	35	3.43	1.93	0.70	5.31	0.203
Partner:						
None (Ref.)	309	27.29	1			
Any	897	72.71	**2.71**	1.68	4.37	<0.001
Report of a lung disease:						
None (Ref.)	1,087	89.6	1			
Any	119	10.40	**1.93**	1.09	3.41	0.024
Difficulty with number of mobility activities:						
None or one (Ref.)	407	32.54	1			
Two	242	19.54	**1.73**	0.99	3.03	0.053
Three or four	267	22.73	**2.56**	1.44	4.55	0.001
Five to seven	214	18.31	**3.92**	2.10	7.36	<0.001
Eight to ten	76	6.88	**3.24**	1.31	8.01	0.011
Difficulty with number of ADLs:						
None (Ref.)	753	61.34	1			
One	272	23.14	**2.07**	1.35	3.16	0.001
Two or three	141	12.05	**2.77**	1.48	5.16	<0.001
Four to six	40	3.47	**3.18**	1.05	9.64	0.041
Difficulty with number of IADLs:						
None (Ref.)	754	61.84	1			
One	251	20.64	**6.25**	4.12	9.47	<0.001
Two to four	168	14.70	**13.43**	7.62	23.68	<0.001
Five to nine	33	2.83	**31.00**	7.83	122.75	<0.001
Reporting limiting long-standing illness:						
No (Ref.)	544	44.50	1			
Yes	662	55.50	**2.07**	1.36	3.15	<0.001
Home with adaptation (*e.g.* hand rails):						
No (Ref.)	748	59.90	1			
Yes	458	40.10	**0.66**	0.44	0.98	0.040
Currently using a cane/walking stick:						
None (Ref.)	793	64.23	1			
Any	413	35.77	**1.74**	1.17	2.61	0.007
Receiving any paid-for care:						
No (Ref.)	1,164	96.45	1			
Yes	42	3.55	**0.33**	0.15	0.71	0.005
Currently using occupational therapy/physiotherapy:						
No (Ref.)	1,135	94.38	1			
Yes	71	5.62	**2.38**	1.28	4.43	0.006

*Notes*: N=1,206. CI: confidence interval. Ref.: reference category. ADLs: activities of daily living. IADLs: instrumental activities of daily living.

*Source*: English Longitudinal Study of Ageing wave 4, authors' calculations.

*Significance*: Significant results are shown in bold.

### The determinants of receiving informal support for women (final model)

Among women, the strongest independent determinants of receiving informal support include marital status, whether or not children are living in the household, the household wealth, as well as the report of difficulty with cognitive, mobility or IADL tasks, and the receipt of professional support. Being married among women shows the strongest association with being in receipt of informal support compared to other groups of marital status, and living in households in the lowest wealth quintile is a stronger predictor of receiving informal support than living in households in the fourth wealth quintile (Table 2). Similarly to men, a difficulty with a high number of IADLs is the strongest predictor of women receiving informal support. For example, the odds of receiving informal support among women who are experiencing difficulty with two to four IADLs are almost 18 times those of women who are experiencing no difficulty, while the odds among women who are experiencing difficulty with five to nine IADLs are ten times those of women who are experiencing no such difficulty. Table 2 also indicates that among women who have been diagnosed with dementia, the odds of receiving informal support are almost 15 times the odds among women who have not been diagnosed with this condition. Finally, women who are receiving paid-for support are less likely to be receiving informal support than women who are not receiving paid-for support.

**Table 2.** The determinants of receiving informal support for women (final model)

	Unweighted N (base sample)	Weighted %	Odds ratio	Lower 95% CI	Upper 95% CI	*p*
Legal marital status:						
Single never married (Ref.)	95	4.53	1			
Married or civil partnered	857	43.07	**12.78**	5.54	29.53	<0.001
Separated or divorced	172	7.74	**2.75**	1.08	7.00	0.034
Widowed	780	44.67	**3.89**	1.71	8.86	0.001
Children in household:						
None (Ref.)	1,718	89.35	1			
Yes	186	10.65	**1.98**	1.28	3.07	0.002
Benefit unit equivalised wealth quintile:						
Poorest quintile (Ref.)	463	27.71	1			
Second-poorest quintile	416	21.71	1.08	0.74	1.58	0.695
Middle quintile	415	20.70	0.75	0.50	1.11	0.151
Second-richest quintile	342	17.29	**0.57**	0.36	0.88	0.012
Richest quintile	268	12.59	0.68	0.43	1.07	0.095
Number of errors in orientation and time:						
No errors (Ref.)	1,423	73.02	1			
One error	382	20.74	1.22	0.88	1.70	0.228
Two or more errors	99	6.24	**1.98**	1.01	3.88	0.046
Diagnosed with a type of dementia:						
No (Ref.)	1,890	99.00	1			
Yes	14	1.00	**15.34**	1.32	178.72	0.029
Number of mobility activities has difficulty with:						
None or one (Ref.)	416	20.9	1			
Two	380	19.09	**2.07**	1.27	3.37	0.004
Three or four	468	24.21	**4.07**	2.55	6.49	<0.001
Five to seven	462	25.66	**4.86**	2.97	7.93	<0.001
Eight to ten	178	10.13	**6.21**	3.32	11.59	<0.001
Number of IADLs has difficulty with:						
None (Ref.)	1,105	55.24	1			
One	388	20.72	**4.54**	3.25	6.33	<0.001
Two to four	363	20.83	**17.58**	11.75	26.31	<0.001
Five to nine	48	3.22	**9.65**	3.65	25.46	<0.001
Difficulty with walking a quarter of a mile:						
No difficulty (Ref.)	871	42.39	1			
Some difficulty	405	21.04	**1.76**	1.23	2.53	0.002
Much difficulty	219	12.47	**2.25**	1.45	3.49	<0.001
Unable to do this	409	24.10	**2.37**	1.55	3.63	<0.001
Receiving any paid-for care:						
No (Ref.)	1,749	91.99	1			
Yes	155	8.01	**0.64**	0.40	1.02	0.061
Currently using chiropodist:						
No (Ref.)	1,171	60.11	1			
Yes	733	39.89	**1.32**	1.00	1.75	0.053
Currently using exercise therapy:						
No (Ref.)	1,699	90.43	1			
Yes	205	9.57	**0.49**	0.29	0.82	0.007

*Notes*: N=1,904. CI: confidence interval. Ref.: reference category. IADLs: instrumental activities of daily living.

*Source*: English Longitudinal Study of Ageing wave 4, authors' calculations.

*Significance*: Significant results are shown in bold.

### The determinants of receiving state support

Table 3 shows the final model for the determinants of receiving state support for both men and women. The number of ADLs and IADLs one has difficulty with are the strongest predictors of receiving state support, for example among people who have difficulty with four to six ADLs, the odds of receiving state support are three times the odds among people experiencing no such difficulty. In line with these results, the odds of receiving state support for a person who has ‘much difficulty’ in walking a quarter of a mile or is unable to do this are between two and six times the odds of a person who does not have a difficulty with this task. The strong gradient reflected in the number of both ADLs and IADLs, and in the difficulty in walking a long distance, indicates an older person's increased likelihood of being eligible for, and accessing, state support as their functionality is impaired and their needs increase. Table 3 also shows that being single is more strongly associated with receiving state support than being married or widowed. Finally, the use of a wheelchair, a personal alarm and any other care are strongly associated with the receipt of state support, while the use of a walking stick is not.

**Table 3.** The determinants of receiving state support (final model)

	Unweighted N (base sample)	Weighted %	Odds ratio	Lower 95% CI	Upper 95% CI	*p*
Legal marital status:						
Single never married (Ref.)	161	4.41	1			
Married or civil partnered	1,874	53.33	**0.17**	0.06	0.48	0.001
Separated or divorced	287	7.58	0.36	0.10	1.28	0.116
Widowed	1,073	34.68	**0.36**	0.13	0.99	0.048
Access to a car:						
No (Ref.)	853	28.35	1			
Yes	2,542	71.65	**0.57**	0.33	0.99	0.045
Number of ADLs has difficulty with:						
None (Ref.)	2,145	61.25	1			
One	665	20.17	1.53	0.63	3.73	0.352
Two or three	436	13.6	**3.01**	1.24	7.37	0.015
Four to six	149	4.98	**3.85**	1.43	10.41	0.008
Number of IADLs has difficulty with:						
None (Ref.)	2,145	55.73	1			
One	665	20.26	**5.94**	1.12	31.58	0.037
Two to four	436	18.65	**14.50**	2598	81.06	0.002
Five to nine	149	5.37	**21.87**	3.66	130.57	<0.001
Difficulty with walking a quarter of a mile:						
No difficulty (Ref.)	1,537	42.27	1			
Some difficulty	741	22.03	1.51	0.31	7.26	0.607
Much difficulty	402	12.54	**3.01**	0.65	14.01	0.161
Unable to do this	714	23.16	**6.52**	1.48	28.67	0.013
Currently using a cane/walking stick:						
No (Ref.)	2,226	63.58	1			
Yes	1,168	36.42	**0.40**	0.24	0.67	0.001
Currently using a manual/electric wheelchair:						
No (Ref.)	3,188	93.33	1			
Yes	206	6.67	**2.43**	1.32	4.49	0.005
Currently using a personal alarm:						
No (Ref.)	3,206	93.44	1			
Yes	188	6.56	**2.08**	1.03	4.20	0.042
Receiving any other care:						
No (Ref.)	3,310	97.21	1			
Yes	83	2.79	**1.96**	0.86	4.43	0.107

*Notes*: N=3,395. CI: confidence interval. Ref.: reference category. ADLs: activities of daily living. IADLs: instrumental activities of daily living.

*Source*: English Longitudinal Study of Ageing wave 4, authors' calculations.

*Significance*: Significant results are shown in bold.

The receipt of paid-for support by older men and women is associated most strongly with the number of IADLs a person had difficulty with. For example, the odds of receiving ‘privately paid-for support’ for a person who reports having difficulty with one IADL are almost 42 times those of a person who reports having no difficulty with IADLs (Table 4), although the interpretation of this result requires caution as the confidence intervals between some categories overlap. Beyond this strong effect, gender and marital status are strong predictors of receiving paid-for support, with women being more strongly associated with this source of support than men, and single never married people being more strongly associated than other categories of marital status. The odds of receiving paid-for support among older people who are living with their child(ren) are 0.22 of the odds of people who are not living with their children, while a person's health status is for the first time associated with the receipt of support from a particular source, with the odds of being in receipt of paid-for support being almost double among those reporting a LLSI compared to those who do not report a LLSI. Living in the poorest households is negatively associated with the receipt of paid-for support, and receiving paid-for support is uniformly associated with richer households except for households in the second-richest quintile. Finally, the incorporation of home adaptations, the use of a personal alarm, occupational therapy or chiropody, and visiting a lunch club, are all positively associated with the receipt of paid-for support by older people.

**Table 4.** The determinants of receiving paid-for support (final model)

	Unweighted N (base sample)	Weighted %	Odds ratio	Lower 95% CI	Upper 95% CI	*p*
Gender:						
Men (Ref.)	1,215	38.75	1			
Women	1,922	61.25	**1.63**	1.08	2.47	0.018
Legal marital status:						
Single never married (Ref.)	145	4.31	1			
Married or civil partnered	1,724	53.06	**0.29**	0.14	0.62	0.002
Separated or divorced	266	7.63	0.83	0.34	2.06	0.705
Widowed	1,002	0.35	0.95	0.46	1.99	0.898
Children in household:						
None (Ref.)	2,815	88.79	1			
Any	322	11.21	**0.22**	0.08	0.56	0.002
Benefit unit equivalised wealth quintile:						
Poorest quintile (Ref.)	728	26.46	1			
Second-poorest quintile	659	21.01	**2.52**	1.46	4.23	<0.001
Middle quintile	650	20.10	**2.85**	1.61	4.94	<0.001
Second-richest quintile	613	18.54	**4.12**	2.30	7.20	<0.001
Richest quintile	487	13.89	**3.27**	1.54	6.81	0.002
Number of IADLs has difficulty with:						
None (Ref.)	1,873	57.71	1			
One	644	20.70	**41.86**	18.95	91.69	<0.001
Two to four	538	18.51	**26.05**	11.54	58.85	<0.001
Five to nine	82	3.08	**30.29**	10.67	86.87	<0.001
Report of limiting long-standing illness:						
No (Ref.)	1,451	45.73	1			
Yes	1,686	54.27	**2.76**	1.71	4.49	<0.001
Home with an adaptation (*e.g.* hand rails):						
No (Ref.)	1,853	56.71	1			
Yes	1,284	43.29	**1.79**	1.20	2.63	0.004
Currently using a personal alarm:						
No (Ref.)	2,975	93.85	1			
Yes	162	6.15	**2.03**	1.18	3.44	0.010
Have ever attended a lunch club:						
No (Ref.)	3,056	97.12	1			
Yes	81	2.88	**2.53**	1.15	5.50	0.020
Currently using occupational therapy/physiotherapy:						
No (Ref.)	2,917	93.28	1			
Yes	220	6.72	**2.50**	1.52	4.07	<0.001
Currently using chiropodist:						
No (Ref.)	2,092	61.17	1			
Yes	1,045	34.83	**1.83**	1.28	2.62	0.001

*Notes*: N=3,137. CI: confidence interval. Ref.: reference category. IADLs: instrumental activities of daily living.

*Source*: English Longitudinal Study of Ageing wave 4, authors' calculations.

*Significance*: Significant results are shown in bold.

## Discussion and conclusion

To date, the academic literature has often discussed the relationship between an individual's health status, reflected in the report of difficulty with different activities, and the amount of support received in later life (Lin and Wu 2011). Building on this literature, the goal in our paper has been to incorporate demographic and socio-economic characteristics in this analysis, and to examine the predictors of receiving support from different sources (informal, paid-for, state), and to our knowledge, this is the first study of this kind. In line with existing research, the analysis shows that the report of difficulty with activities and mobility increases in line with age for both men and women (Breeze and Stafford 2010). Although informal support constitutes the cornerstone of care provided to older people, this research has shown that significant proportions of older people who report difficulties are also in receipt of statutory and paid-for support. Understanding the factors associated with the receipt of support from different sources in later life is a key step in the design and monitoring of social care, and is critical at a time of demographic and policy changes which can adversely affect older people's receipt of support (Vlachantoni *et al.* 2011).

The research in this paper shows three key findings which have significant implications for the organisation of social care support for older people. Firstly, the receipt of support in later life, from any source, is primarily determined by the number of IADLs, and to a lesser extent the number of ADLs, a person has difficulty with. For example, in the models explaining the receipt of support from different sources, the odds of receiving support among people who report a difficulty with five to nine IADLs are between 22 and 30 times the odds among people who report no such difficulty. The only exception to this pattern is women who are in receipt of informal support, where this effect is smaller but still significant. This kind of difficulty may in turn interact with a person's demographic and health characteristics, as well as socio-economic characteristics, to affect their receipt of support from different sources. The effect of certain demographic characteristics in the final models reflects this relationship, *e.g.* a person's marital status and the presence of children in the household are important determinants of women's receipt of informal support and the receipt of paid-for support by both men and women, while marital status is a key determinant of receiving state support by both men and women. Although the bivariate analysis shows that people in the older age groups are more likely to receive support than younger old people, age and health in the multivariate analysis are mostly accounted for by a person's difficulty with ADLs, IADLs or mobility tasks, and for this reason are not shown to have a significant association with the receipt of support from different sources. There are two exceptions to this rule; firstly, the model for the receipt of informal support by men shows that men aged 75–84 are more likely to receive such support than men aged 65–69, and the lack of significance for the older age groups may be due to low cell counts, or more likely, the overwhelming effect of reporting difficulty with different activities, which is more important than an older person's age *per se*. Secondly, the model for the receipt of paid-for support shows that the report of a LLSI is a strong determinant for both men and women. Finally, the association between the use of a wheelchair or a personal alarm and the receipt of state support may reflect statutory provision towards individuals with a high level of support needs, whereas the use of a walking stick, which is not associated with state support, may indicate an individual's ability to purchase it privately and/or a lower level of support need.

Beyond the strength of the IADL and ADL variables across all models, the analysis shows that different factors are associated with the receipt of support from different sources, and there are key gender differences in this respect. For example, variables related to one's physical health status are only significant determinants of men's receipt of informal support, while variables related to one's mental health status are part of the explanation for women's receipt of informal support. To some extent, such differences may reflect gender differences in the prevalence of particular physical or mental conditions among older men and women. Crucially, marital status or partnership is an important determinant for the receipt of support from any source, reflecting the role of spousal care in later life among both men and women (Dahlberg, Demack and Bambra 2007). Finally, the use of services such as occupational therapy, exercise therapy or the services of a chiropodist are associated with the receipt of informal or paid-for support, while the use of aids such as a wheelchair or a personal alarm are more closely associated with the receipt of state support. From the perspective of policy design, the cost of providing state support, such as aids, may be significantly higher when catering for individuals with high-support needs compared to individuals with low-support needs, even if the absolute number of such individuals is relatively small (Humphries, Forder and Fernandez 2010).

The third key finding in this paper is that different kinds of needs are associated with the receipt of support from different sources, and this is reflected in both the bivariate and multivariate analyses. The results show that the receipt of informal and state support is associated with a person's difficulty with ADLs such as bathing and getting dressed, while the receipt of paid-for support is more closely associated with one's difficulty with specific IADLs, such as shopping and doing housework or garden work. These bivariate results are in line with multivariate results on the stronger association between the number of IADLs and the receipt of formal paid-for support, compared to the association between the number of IADLs and the receipt of informal or formal state support. This result may indicate that formal paid-for support is easier to access for support with such tasks as shopping for groceries, or doing work around the house or garden, and the stronger effect for those being in the two richest quintiles of the income distribution may reflect that individuals receiving such support belong to a group of a higher socio-economic status. In terms of policy design, such results reaffirm the importance of the state's contribution to support for older people, particularly with ADLs which are fundamental for functioning on a daily basis, such as bathing and dressing. On the other hand, support of older people with IADLs, such as shopping or housework, which is more closely associated with the informal and the paid-for sectors, may be more effectively organised through community volunteer initiatives rather than formal state services.

Our results are also indicative of the extent to which support from one source can complement or substitute support from a different source, however, such patterns are different depending on the specific task, and in line with existing research (Mentzakis, McNamee and Ryan 2009). Although this paper did not set out to address the question of ‘substitutability’ of support between different sources, this result offers indicative evidence that there is some degree of substitution between different sources of support for older people. For example, the receipt of informal support is associated with a decreased likelihood of receiving paid-for support for both men and women, and this effect is greater among women. Similarly, the negative association between the receipt of informal support by women and living in a household in the second-richest wealth quintile may reflect the financial capacity of older women to rely on support from sources other than their relatives or friends. This is consistent with research showing that the absence of informal support may be a reason why older people seek paid-for support, if they can afford it, or are eligible for state support (Breeze and Stafford 2010). Variables indicating a higher socio-economic status, such as one's access to a car and living in a household in the richer wealth quintiles, are associated with a lower likelihood of receiving informal support by women, a lower likelihood of receiving state support by men and women, and a higher likelihood of receiving paid-for support by men and women. Although a person's difficulty with performing ADLs or IADLs, and therefore a person's need, remains by far the strongest determinant of receiving support from any source, such associations point towards the relative importance of a person's socio-economic status in having their needs met, particularly in relation to IADLs. Such results need to be considered alongside the proportion of unmet need for particular tasks; evidence from the same dataset published elsewhere show that about one-third of older people who report a difficulty with dressing and bathing receive no help from any source (Vlachantoni *et al.* 2011).

We used the number of activities an individual reports difficulty with as a measure of the type and the level of difficulty experienced by the individual, thereby creating a range of predictors for the receipt of social support from different sources. The paper does not attempt to create a hierarchy of difficulty or need, *e.g.* claiming that one older person's difficulty with three IADLs is equivalent to another older person's difficulty with a single ADL. Rather, the focus of the paper is on understanding the role played by an older person's individual characteristics, and their report of difficulty, in their receipt of social care support from different sources. At least three weaknesses in this paper's methodological approach should be acknowledged. The first relates to the cross-sectional nature of the data, which prevents us from understanding the impact of changes in individual characteristics and report of difficulty on their receipt of social care support from different sources, but which we see as the aim of a separate research endeavour. The second refers to the predictors of difficulty with activities and mobility tasks, the way this question is asked in the ELSA questionnaire and the fact that it produces a binary response (yes/no) rather than a gradient of difficulty (*e.g.* a little or a lot of difficulty). To some extent, this weakness is mediated by our derived variables adding the number of difficulties reported within the ADL, IADL and mobility categories. The third relates to attrition and the ways in which it has affected our analytical sample. The ELSA team's research showed that non-respondents were different to respondents in terms of their housing tenure, ethnicity, highest educational qualification and marital status (Cheshire *et al.* 2012). The use of cross-sectional weights in the analysis ‘corrects’ the sample by taking into account sample stratification and non-response. However, Banks, Muriel and Smith (2011), who compared the impact of attrition on estimates of disease prevalence in the Health and Retirement Survey (for the USA) and the ELSA, found that for the 70–80-year-old group, attrition was negatively associated with high educational status and numerical ability in the previous wave. In the context of our analysis, this may mean that higher-educated persons are over-represented in the 70–80 age group, which, in the light of existing literature discussed before, may in turn contribute to an over-representation of higher socio-economic status and the tendency to receive support from ‘paid-for’ sources in this age group. A final note of caution relates to the overlap in the confidence intervals between the categories of certain key predictors, such as the report of difficulty with ADLs or IADLs, for the receipt of support from different sources. Such overlap needs to be taken into account when interpreting the results.

The results in this paper contribute to our understanding of the factors associated with the receipt of social support, and raise critical issues about the organisation of social care provision in England and beyond. Policy concerns about the supply of social care increasingly refer to the ‘mix’ between informal, formal state and formal paid-for sources of support, and the need to strike a balance between public and private resources in the context of an ageing population (Organisation for Economic Co-operation and Development (OECD) 2011). The strength of the report of difficulties with ADLs, IADLs and/or mobility tasks as predictors for the receipt of support from any source confirms the link between physical and mental health status, and the receipt of support from different sources. For policy makers in the developed world, it is our further understanding of particular tasks associated with formal state support, as well as with informal and formal paid-for support, which can directly inform decisions about the design of social care services. Further analysis of the relationship between specific types of difficulties and the receipt of support can shed more light on policies aimed at addressing the needs of those requiring social support.

## NOTES

1 *See Personal Social Services Expenditure and Unit Costs, England* for various years, available online from the NHS Information Centre at www.ic.nhs.uk [Retrieved July 2012].

2 All socio-economic variables refer to the total ELSA sample in 2008.

3 The equivalence scale used here is an OECD equivalence scale and assigns a weight of 0.5 to second adults and dependent children aged 14 and over and a weight of 0.3 to children under 14 years of age. A benefit unit is defined as a single person or a couple (regardless of whether they keep their finances separate or together). Income includes total income from employment, self-employment, private pensions, state pensions, benefits, assets (*e.g.* interest from savings), while wealth refers to total non-pension wealth, including net housing wealth (*e.g.* house value) and net non-housing wealth (*e.g.* savings).

4 The variable on home adaptations included the following: widened doorways or hallways, ramps or street-level entrances, hand rails, automatic or easy-open doors, accessible parking or drop-off site, bathroom modifications, kitchen modifications, lift, chair lift or stair glide, alerting device or any other special feature.
